# Correlated fluorescence quenching and topographic mapping of Light-Harvesting Complex II within surface-assembled aggregates and lipid bilayers

**DOI:** 10.1016/j.bbabio.2018.06.011

**Published:** 2018-10

**Authors:** Peter G. Adams, Cvetelin Vasilev, C. Neil Hunter, Matthew P. Johnson

**Affiliations:** a School of Physics and Astronomy, University of Leeds, Leeds LS2 9JT, UK; b Astbury Centre for Structural Molecular Biology, University of Leeds, Leeds LS2 9JT, UK; c Department of Molecular Biology and Biotechnology, University of Sheffield, Sheffield S10 2TN, UK

**Keywords:** Light harvesting, Photosynthesis, Non-photochemical quenching (NPQ), Time-resolved fluorescence, Atomic force microscopy (AFM), Light-Harvesting Complex II (LHCII)

## Abstract

Light-Harvesting Complex II (LHCII) is a chlorophyll-protein antenna complex that efficiently absorbs solar energy and transfers electronic excited states to photosystems I and II. Under excess light intensity LHCII can adopt a photoprotective state in which excitation energy is safely dissipated as heat, a process known as Non-Photochemical Quenching (NPQ). In vivo NPQ is triggered by combinatorial factors including transmembrane ΔpH, PsbS protein and LHCII-bound zeaxanthin, leading to dramatically shortened LHCII fluorescence lifetimes. In vitro, LHCII in detergent solution or in proteoliposomes can reversibly adopt an NPQ-like state, via manipulation of detergent/protein ratio, lipid/protein ratio, pH or pressure. Previous spectroscopic investigations revealed changes in exciton dynamics and protein conformation that accompany quenching, however, LHCII-LHCII interactions have not been extensively studied. Here, we correlated fluorescence lifetime imaging microscopy (FLIM) and atomic force microscopy (AFM) of trimeric LHCII adsorbed to mica substrates and manipulated the environment to cause varying degrees of quenching. AFM showed that LHCII self-assembled onto mica forming 2D-aggregates (25–150 nm width). FLIM determined that LHCII in these aggregates were in a quenched state, with much lower fluorescence lifetimes (~0.25 ns) compared to free LHCII in solution (2.2–3.9 ns). LHCII-LHCII interactions were disrupted by thylakoid lipids or phospholipids, leading to intermediate fluorescent lifetimes (0.6–0.9 ns). To our knowledge, this is the first in vitro correlation of nanoscale membrane imaging with LHCII quenching. Our findings suggest that lipids could play a key role in modulating the extent of LHCII-LHCII interactions within the thylakoid membrane and so the propensity for NPQ activation.

## Highlights

• Light-Harvesting Complex II can be manipulated & quenched on mica surfaces, cf. NPQ.
• LHCII assembles into small 2D aggregates on mica, shown by Atomic Force Microscopy.
• LHCII aggregates are highly quenched as shown by Fluorescence Lifetime Microscopy.
• Lipids caused rearrangement of LHCII and an intermediate level of quenching.
• Correlation of AFM and FLIM is a powerful strategy to link structure and function.

## Graphical abstract

**Figure:**
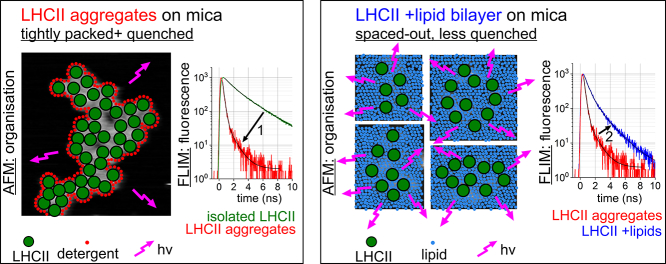

## 1 Introduction

Photosynthetic light harvesting occurs at the thylakoid membranes of chloroplasts of plants, algae and cyanobacteria [1]. Light-harvesting (LH) membrane proteins act as a non-covalent scaffold for precise orientation of embedded chlorophyll (Chl) and carotenoid pigments. Absorption of a photon of sunlight by a pigment molecule promotes an electron to a higher-energy excited state, which can either decay to its ground state via fluorescence, or this energy can be non-radiatively transferred between pigments or dissipated by some other mechanism. In photosynthetic membranes, excitation energy is efficiently transferred within and between LH complexes to the photosynthetic reaction centre (RC) complexes, wherein photochemical charge separation traps energy in a chemical form (reviewed in [2]). Light-Harvesting Complex II (LHCII) is formed of a trimer of Lhcb1, 2 and 3 proteins in different combinations [3], each of which binds 8 Chl *a*, 6 Chl *b* and 4 carotenoid pigments [4, 5]. In plants, LHCII acts as an efficient antenna system for both photosystem I and II (PSI and *PSI*I) in low light [6, 7], however in excess light it absorbs more solar energy than can be utilised for photosynthesis. Light saturation of photosynthesis can prolong the excited state lifetime of Chl and so increase the probability of Chl triplet formation and so singlet oxygen production, causing photoinhibitory damage to the RCs (reviewed in [8]). In mitigation, plants have evolved a photoprotective mechanism, known as non-photochemical quenching (NPQ) of Chl fluorescence, that leads to the safe dissipation of excess absorbed excitation energy in LHCII as heat (reviewed in [9]). NPQ is triggered under excess light conditions by the interplay of three factors: the transmembrane ΔpH [10], the PsbS protein [11] and the enzymatic de-epoxidation of the LHCII bound xanthophyll violaxanthin to zeaxanthin [12]. Together these factors bring about a change in conformation of LHCII, and possibly the minor LHC antenna complexes CP24, 26 and 29 [[13], [14], [15]], that leads to formation of dissipative energy transfer pathways correlated to a dramatic shortening of the Chl excited state lifetime [16, 17]. The dynamics and efficiency of NPQ formation and relaxation have been shown to be crucial to plant fitness in fluctuating light environments as experienced in nature [[18], [19], [20]].

NPQ was shown using freeze-fracture electron microscopy to be accompanied by increased LHCII-LHCII interactions and a remodelling of the thylakoid membrane in spinach chloroplasts, changes that were reversed upon lowering the light intensity [[21], [22], [23], [24]]. These increased LHCII-LHCII interactions were dependent on the presence of PsbS and ΔpH and were enhanced by the de-epoxidation of violaxanthin to zeaxanthin [22]. These changes in LHCII organisation in vivo bear similarity to those observed following induction of quenching in vitro in purified LHCII by manipulation of the detergent/protein or lipid/protein ratio and/or pH, which also lead to aggregation of LHCII [[25], [26], [27]]. The system of LHCII aggregates has thus become a useful model for studying NPQ in vitro. In recent years it has been shown that whilst LHCII-LHCII interactions promote quenching they are not essential, since switching between quenched and nonquenched states is observed in isolated LHCII complexes covalently bound to glass [28], suspended in gels [29] and indeed in single molecule measurements of isolated complexes bound to a substrate [30, 31] or held in solution electrokinetically without chemical modification [32]. In each case, the quenching was fully reversible and shared many of the same spectroscopic features as NPQ in vivo, including distortion of bound Chl, lutein and neoxanthin pigments [33, 34] and red-shift of the terminal emitter Chls in LHCII [35, 36]. The photophysical mechanism of NPQ within LHC complexes remains under debate with both chlorophyll-lutein [34], chlorophyll-zeaxanthin [37] and chlorophyll-chlorophyll [38] interactions being implicated.

Whilst studies on purified LHCII in vitro have revealed many details regarding NPQ, it is unclear how LHCII-LHCII interactions may be modified by lipids [39], potentially a crucial factor in modulation of NPQ in vivo. Studies using proteoliposomes have shown a dramatic quenching of LHCII as the lipid-to-protein ratio is decreased suggestive of LHCII clustering [27, 40, 41], however this solution-based system is not conducive to visualization of nanoscale protein organisation. In principle, a simple well-controlled in vitro system that can be imaged under liquid by high resolution microscopies could shed light on such aspects of NPQ control. Herein, we describe such a system by the binding of purified LHCII (with or without a lipid bilayer) to an optically transparent and atomically flat mica substrate that is amenable to nanoscale topographical mapping by atomic force microscopy (AFM) and functional imaging by fluorescence lifetime microscopy (FLIM). This allows us to correlate the organisation of just a few LHCII complexes to their exciton dynamics of relevance to NPQ.

## 2 Materials and methods

### 2.1 Biochemical protein purification and characterisation

All chemicals were purchased from Sigma-Aldrich (St. Louis, MO) unless otherwise specified. Extraction and purification of LHCII from spinach leaves followed a protocol adapted from Johnson and co-workers [35]. Spinach leaves (purchased from a local supermarket) were added to ice-cold Preparation medium (300 mM sucrose, 5 mM EDTA, 50 mM HEPES, pH 7.5), homogenized in a food blender and liquid recovered after filtration through muslin cloth. Chloroplasts were collected by centrifugation (3000 ×*g*, 15 min, 4 °C), resuspended in Break medium (5 mM EDTA, 10 mM Tricine pH 7.4) and osmotically burst with an equal volume of Lysis medium (400 mM sucrose, 5 mM EDTA, 10 mM Tricine, pH 7.4). Thylakoid membranes were collected by centrifugation as above and the pellet washed with high EDTA and high NaBr buffers. Thylakoids were adjusted to 0.5 mg Chl/mL and then solubilized using a final concentration of 1.0% α-DDM in a 20 mM HEPES buffer for 1 h on ice. LHCII was isolated on continuous gradients of 8–14% sucrose (in 20 mM HEPES pH 7.5 and 0.03% α-DDM), via ultracentrifugation at 100,000 ×*g* for 36 h at 4 °C. The green band at 12% sucrose representing mostly trimeric complexes was harvested. LHCII was further purified by subsequent size-exclusion chromatography in 150 mM NaCl, 0.03% α-DDM, 20 mM HEPES (pH 7.5) at a flow rate of 0.3 mL/min using a 16/600 Superdex 200 prep grade column on an AKTA Prime FPLC system (GE Healthcare Life Sciences, PA, USA). Sucrose or salts were diluted and the protein concentrated as necessary between stages and finally, using 30 kDa Amicon Ultra centrifugal filters (Merck Millipore, UK). Protein purity was assessed via denaturing SDS-PAGE with Coomassie or Sypro Ruby staining and protein oligomeric state was assessed via non-denaturing Clear Native PAGE with Coomassie staining, using pre-made gels (NuPAGE Novex 12% Bis-Tris and NativePAGE Novex 4–16% Bis-Tris, respectively, from ThermoFisher Scientific) and pre-stained protein ladder (BioRad, CA, USA).

### 2.2 Sample preparation for microscopy

Substrates for all microscopy were thinly cleaved mica glued to glass coverslips with an optically-transparent adhesive (Norland Products, NY), unless otherwise stated. Samples were prepared on mica with variations as described in the text. Generally, purified trimeric LHCII (approx. 10 μM) suspended in 0.03% α-DDM was diluted in detergent-free Buffer A (20 mM HEPES, pH 7.5) to the desired final concentration and then incubated with the freshly-cleaved mica surface for 20 min. Samples were then washed with 10 changes of the desired imaging buffer and LHCII aggregates could be imaged at this stage. For experiments with lipids, samples were further incubated with lipid vesicles (0.5 mg/mL) for 20 min and then washed with 10 changes of the imaging buffer. For AFM, the imaging buffer was 20 mM HEPES (pH 7.5) and the sample was imaged in an open droplet. For FLIM, the imaging buffer was sparged with N_2_ gas to displace O_2_ and additionally contained the oxygen-scavenging enzyme system of 2.5 mM protocatechuic acid and 50 nM protocatechuate-3,4-dioxygenase from *Pseudomonas* species [42] and then the sample was sealed with a glass slide to confine an aqueous volume.

Lipids in dry form were purchased from Avanti Polar Lipids (Alabaster, AL) except the Texas Red DHPE lipid dye (ThermoFisher Scientific). For the model phospholipid system the lipids used were 100% DOPC. For the model thylakoid system, a mixture similar to those previously described as natural ratios [43] was adapted for use with planar surfaces as described by Gräb and co-workers [44], comprising: 35% MGDG, 20% DGDG, 12% SQDG, 8% Soy PG, 25% DOPC (w/v%), for further methodological details see the Supplementary information. Lipids were dissolved in 5:1 chloroform: methanol and mixed to the desired ratios in glass vials in and dried under nitrogen flow for 30 min and then in a vacuum desiccator overnight. Lipids were hydrated in buffer of choice (20 mM HEPES pH 7.5 for DOPC or 150 mM NaCl, 20 mM MES pH 6.5 for thylakoid lipids) to 1 mg/mL and vesicles prepared by probe sonication at 4 °C.

### 2.3 Atomic force microscopy

AFM was performed under the described aqueous buffers using a Dimension FastScan and FastScan D type probes (Bruker Nano Surfaces Division). Parameters were optimized whilst imaging to minimize applied forces, at low tapping amplitudes and moderate gains, typically scanning at 1–4 Hz and 1024 × 1024 pix. Topographs were processed and analysed using Nanoscope Analysis software (v1.8).

### 2.4 Absorption and fluorescence spectroscopy and microscopy

Cuvette-based UV–Vis absorption spectra were acquired using an Agilent Cary 5000 spectrophotometer. LHCII trimer molar concentration was estimated from absorption spectra (where A (675) of 1.0 is 400 nM). Cuvette-based steady-state and time-resolved fluorescence spectra were acquired using an Edinburgh Instruments FLS980 spectrophotometer. FLIM was performed on a home-built inverted optical microscope as previously described [28] equipped with a spectrometer (Acton 150, Princeton Instruments) and an electron-multiplying charge-coupled device (EMCCD) camera (ProEM 512, Princeton Instruments). Excitation light was filtered by a 472/30 nm bandpass filter and reflected towards the sample using a dichroic beamsplitter, emission was passed through the same dichroic towards further emission filters and the desired detector. For simple fluorescence imaging the excitation source was a collimated LED and emission was collected through a 679/41 nm bandpass filter by the EMCCD. For fluorescence emission spectra and time-resolved measurements, the excitation source was a LDH 485 nm laser (PicoQuant GmbH) which was focused to a diameter of ~800 nm and positioned so that the laser spot was at a selected region of the sample. The laser was driven by a PDL 828 Sepia II burst generator module (PicoQuant GmbH) operated at a repetition rate of 0.5 MHz with pulse FWHM of ~50 ps. Laser power was set at between 54.7% and 100% and an ND filter used to modulate excitation power as desired. We trialled a series of different excitation power settings (see Supplementary Fig. S1) with estimated fluence at the sample surface of 0.005 to 2.9 mJ/cm^2^ (pulse energy of 0.03 to 14 pJ = 10^13^ to 10^16^ photons/pulse/cm^2^). These control experiments carefully adjusting the excitation fluence show that singlet-singlet annihilation affects the magnitude of lifetimes but do not cause the changes in lifetimes observed due to lipids [45]. A moderate fluence (0.39 mJ/cm^2^) and low fluence (0.05 mJ/cm^2^) were used subsequently. Note, LHC-II trimers under excess detergent and on a Teflon-coated glass substrate (to prevent protein-surface association) has 〈*τ*〉 = 3.9 ns, consistent with previously published data for non-quenched LHCII [29]. No significant photo-bleaching was observed by microscopy. Emission spectra were captured with a slit width of 500 μm and a 150 line mm^−1^ grating at spectrometer central wavelength of 680 nm, collected through a 594 nm longpass filter by the EMCCD. Time-resolved spectra were captured with a slit width of 500 μm and a 300 line mm^−1^ grating at spectrometer central wavelength of 682 nm, collected through a 679/41 nm bandpass filter and focusing slits (effective bandwidth ~5 nm) by a photomultiplier tube (PMT) detector. Timing electronics were a time-correlated single-photon counting (TCSPC) module (SPC-150, Becker & Hickl). Each fluorescence image and the spectra were averages of 8 frames with 0.25 s exposure time; time-resolved measurements generally had 4 s collection time. Analysis of fluorescence decay curves was performed using TRI2 software (v2.8.5) fitting to a bi-exponential function, reconvoluting with the measured IRF, and minimizing residuals and chi^2^ to <1.1. All graphs were generated using OriginPro (v9.1) and figures produced using Xara Photo & Graphic Designer.

## 3 Results

All relevant raw and analysed data are freely available in an online repository [46].

### 3.1 Purification of LHCII trimers

**Fig. 1:**
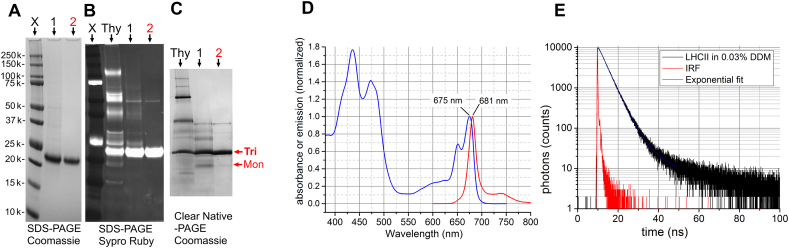
Purification of LHCII trimers. Standard denaturing SDS-PAGE of LHCII samples either with Coomassie staining (A), or more sensitive Sypro Ruby fluorescent staining (equivalent detection to silver staining) (B), or the non-denaturing Clear Native PAGE (C). Pre-stained protein standards (*X*), crude thylakoid membranes after α-DDM solubilisation (*Thy*), LHCII after isolation on sucrose density gradients (*1*), LHCII trimers after addition purification by size exclusion FPLC chromatography (*2*). The position of the band expected to represent trimeric or monomeric LHCII is indicated (*Tri* or *Mon*, respectively). (D) Cuvette-based absorption spectrum (*blue*) and overlaid fluorescence emission scan (*red*) of LHCII at 10 nM LHCII (in 0.03% α-DDM, 20 mM HEPES, pH 7.5). Excitation at 473 nm with slit width = 4 nm, emission slit width = 1 nm. (E) Cuvette-based time-resolved fluorescence measurement of the same LHCII sample produced a fluorescence decay curve as shown, average lifetime = 3.85 ns (see Table 1). Excitation with 473 nm pulsed laser, emission slit width = 1 nm. (For interpretation of the references to color in this figure legend, the reader is referred to the web version of this article.)

LHCII was prepared following well-established protocols modified to give a high purity of trimers (see Methods section). Briefly, thylakoids were extracted from spinach leaves, digested using α-DDM and LHCII trimers were isolated by sucrose gradient sedimentation. SDS-PAGE reveals that LHCII had a reasonable purity after the sucrose gradient stage (one strong band with Coomassie staining, Fig. 1A) and indeed material of this purity has often been used in previous studies of LHCII aggregates [28]. The trimeric form of LHCII was further purified by subsequent high-resolution size-exclusion chromatography. Minor contaminants observed in the sucrose gradient sample with a high sensitivity Sypro Ruby stain were almost entirely removed via the chromatography stage (Fig. 1B) and small amounts of LHCII monomers were also removed to produce a final high purity sample where the trimeric state of LHCII was predominant, as shown by non-denaturing PAGE (Fig. 1C). LHCII trimers were optically characterized in a dilute, stable form in detergent solution in standard cuvette-based absorption and fluorescence spectrometers (at 10 nM LHCII in 0.03% α-DDM): an absorbance spectrum with Chl *a* Q_y_ maximum at 675 nm (Fig. 1D), an emission maximum at 681 nm (Fig. 1D) and a fluorescence decay curve fitted to an amplitude-weighted fluorescence lifetime of *τ* = 3.8–4.0 ns (Fig. 1E). These spectroscopy data are characteristic of purified trimeric LHCII in a highly-emissive native state [29, 31].

In subsequent FLIM measurements of LHCII on a surface, we monitor the fluorescence emission spectrum and compare its similarity to the (above) control of native LHCII as an important confirmation that our protein is intact and functional (no protein damage). By preferential excitation of chl *b* and monitoring chl *a*, this fluorescence emission measurement assesses intra-complex chl *b* → chl *a* energy transfer and thus the level of connectivity of LHCII, as described below (and see Supplementary Fig. S2). In the native LHCII, all chlorophylls and carotenoids are highly connected so that energy rapidly equilibrates across the complex [47]. As a result of downhill energy transfer, steady-state fluorescence emission spectra of functional LHCII are dominated by the low-energy “terminal emitter” chl *a* which have a Q_y_ transition at 681 nm, rather than chl *b* (Q_y_ transition at 640–650 nm) [29, 31]. In our measurements of fluorescence emission from surface-bound complexes we selectively excite chl *b* via laser excitation at 485 nm (the chl *b* Soret band at 480 nm, c.f. the chl *a* Soret band at 430 nm) and look for emission >600 nm. Observation of a peak with identical shape and peak wavelength is good evidence that chl *a* and chl *b* are well connected. Thus, by ruling out bulk protein-pigment configurational changes, we consider that changes to the fluorescence decay curves (and fitted lifetimes) are due to switching of some LHCII into quenched states.

### 3.2 Topographic mapping of the in vitro model of LHCII aggregates

**Fig. 2:**
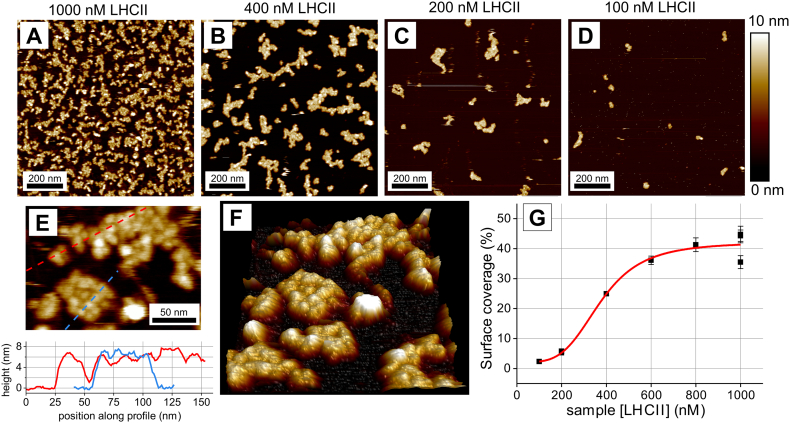
Visualization of the aggregated domains of LHCII by atomic force microscopy. (A)–(D): Representative AFM topographs showing the arrangements formed by deposition of LHCII onto mica substrates for 20 min at a range of LHCII concentration from 1000 to 100 nM. Substrates were washed with buffer prior to imaging. (E) High resolution topograph showing LHCII trimers and height profiles (*below*). (F) 3-D representation of (E). (G) Graph showing the area of the substrate occupied by LHCII (% of total) vs concentration of LHCII applied.

AFM showed that trimeric LHCII self-assembled into domains of varying sizes onto clean, atomically flat mica substrates (Fig. 2; a gallery of further images is shown in Supplementary Fig. S3). Control was exercised over the amount of LHCII deposited on the surface, by varying the concentration of LHCII in the solution incubated with the mica for a constant time before washing away unbound protein (Fig. 2A–D). A qualitative trend was shown where the surface coverage increased with LHCII concentration (Fig. 2G), as may be expected for deposition of self-associating colloids on a surface. High resolution AFM topographs (Fig. 2E–F) reveal domains that contain closely-packed particles with approximate diameters of 10 nm. The maximal height of protrusions above mica was usually ~6 nm, although heights of 7–8 nm are observed, which may be due to packing constraints lifting the protein slightly off the mica. These dimensions are in good agreement with the dimensions of trimeric LHCII from crystal structures [4]. There was no evidence of large 3-D aggregates, which might be expected if extensive aggregation occurs in solution prior to surface association or if LHCII complexes were stacked on the mica substrate. The above observations support the notion that LHCII trimers associate in a process likely to be promoted by and nucleated at the flat mica surface. We expect the lumenal or stromal surface of LHCII interact with the solid surface, because electrostatic attractive interactions between the polar extrinsic residues of LHCII and the mica lattice are more thermodynamically favourable than an interaction between hydrophobic transmembrane segments of LHCII and the mica. From the resolution of our AFM data we cannot distinguish the orientation of LHCII (luminal-side or stromal-side up). We rule out any significant distortion of the complex by means of the fluorescence emission measurements (described later) which provide evidence that the protein is intact and functional.

### 3.3 Fluorescence lifetime imaging demonstrates LHCII aggregates in a highly quenched state

**Fig. 3:**
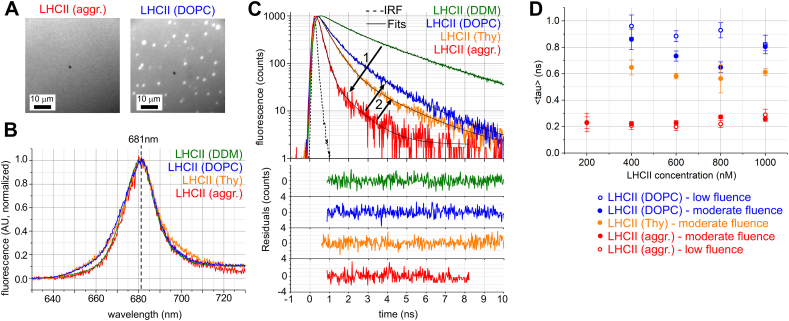
Fluorescence lifetime and spectral imaging microscopy (FLIM) of LHCII on mica under detergent, as aggregates (without lipid), and with lipids. (A) Representative epifluorescence images of LHCII on mica as aggregates, *LHCII* (*aggr*.) (deposited at 1000 nM LHCII trimers for 20 min, washed), or after incubation with lipid vesicles and gentle washing, *LHCII* (*DOPC*) (600 μM lipids for 20 min). Image brightness was increased for *LHCII* (*aggr*.) to be comparable for clarity. (B) Fluorescence emission spectra acquired of representative diffraction-limited regions from each sample. The alternative lipid sample using thylakoid lipid vesicles is denoted as *LHCII* (*Thy*) and the additional control sample of LHCII in detergent solution ~5 μm above mica are also shown, *LHCII* (*DDM*). (C) Picosecond time-resolved fluorescence decay curves of representative regions from samples. Bi-exponential curve fitting (*solid black lines*) and residuals shown, labelled as in (B). The Instrument Response Function (IRF) is shown (*dotted line*), with a measured full-width at half-maximum (FWHM) of ~170 ps. All decay curves normalized to a maximum of 1000 counts (dividing raw data for *DDM*, *DOPC*, *Thy* and *aggr*. by 8.64, 2.44, 2.06 and 1.15, respectively). Arrows represent the proposed *process* (*1*): the deposition of LHCII onto mica and aggregation-induced faster decay, and *process* (*2*): addition of lipids causing limited dis-aggregation and slower decay. Note, only regions of homogeneous fluorescence in observed in *LHCII* (*lipids*) were analysed in (B) and (C) for reproducibility, bright spots were avoided. All samples were measured under N_2_-sparged buffer solution containing the oxygen-scavenging system of PCD/PCA. (D) Graph showing the average fluorescence lifetime 〈*τ*〉 against the concentration of LHCII incubated with mica, as in Table 1.

FLIM was used to measure samples on mica substrates identical to those described above, except that samples were sealed including an enzyme-based oxygen-scavenging system to provide an oxygen-free environment for consistent fluorescence studies (see Methods). Epifluorescence images showed a homogenous level of fluorescence over hundreds of microns (Fig. 3A, *LHCII* (*aggr*.)). The 681–682 nm emission maximum of LHCII aggregates was very similar to the emission maximum of a control sample of LHCII in DDM (Fig. 3B, *LHCII* (*aggr*.) vs *LHCII* (*DDM*)). Thus, deposition of LHCII on mica did not denature or damage the complex, or cause major changes to pigment organisation.

Analysis of the fluorescence decay curves derived from the FLIM data (Fig. 3C) allowed precise quantification of the amount of quenching in each sample. LHCII aggregates on mica had much faster fluorescence decay when compared to LHCII in DDM (Fig. 3C, *arrow 1*). Modelling each decay curve as a bi-exponential decay function produced excellent fitting (chi-squared values <1.1 and low residuals, see Fig. 3C). Lifetime component analysis showed that the amplitude-weighted average lifetime, 〈*τ*〉, was very short at between 0.20 and 0.29 ns for all surface densities measured when compared to LHCII in DDM at 2.22 ns, irrespective of excitation fluence (Table 1, for further data, see Supplementary Fig. S1). Note that the very similar 〈*τ*〉 for all surface densities is consistent with the AFM observation that domain size and therefore the number of connected LHCII did not vary significantly with LHCII concentration, merely the overall surface coverage (i.e. number of domains not their size) (Fig. 2A–D). Further fluorescence decay curves and emission spectra confirming the trends described are shown in Supplementary Figs. S4 and S5. Note that the fluorescence decay of LHCII solution above mica at high concentration (1000 nM) did not change in a sample studied over many hours showing that large aggregates did not form in solution. This somewhat shorter lifetime of 2.2 ns for LHCII diluted with detergent-free buffer compared to the ~4 ns lifetime measured for isolated dilute LHCII in high-detergent solution is probably due to a lower detergent-to-protein ratio [48] which may represent the formation of limited LHCII self-associations in solution prior to nucleation on the mica promoting extensive LHCII-LHCII packing.

**Table 1:** Analysis of FLIM data comparing the fluorescence lifetime of LHCII on mica.

Sample [LHCII] (nM)^a^	Lipids/DDM?	Excitation fluence (mJ/cm^2^)^f^	A1 (%)	*τ*1 (ns)	A2 (%)	*τ*2 (ns)	〈*τ*〉 (ns)	〈*τ*〉 S.D. (ns)
10^b^	DDM^b^	0.39	98	3.73	2	8.96	3.85	N/A
1000^c^	DDM^c^	0.39	49	3.32	51	1.17	2.22	0.04
200	None	0.39	24	0.35	76	0.19	0.23	0.07
400	None	0.39	4	0.70	96	0.21	0.22	0.02
600	None	0.39	2	1.08	98	0.21	0.23	0.02
800	None	0.39	3	1.26	97	0.24	0.27	0.01
1000	None	0.39	2	1.28	98	0.23	0.26	0.02
200	None	0.05	41	0.32	59	0.19	0.23	0.04
400	None	0.05	24	0.30	76	0.19	0.21	0.03
600	None	0.05	14	0.32	86	0.18	0.20	0.03
800	None	0.05	23	0.30	77	0.19	0.22	0.03
1000	None	0.05	29	0.38	71	0.25	0.29	0.04
400	PC lipids^d^	0.39	19	2.04	81	0.60	0.86	0.08
600	PC lipids^d^	0.39	16	1.68	84	0.56	0.73	0.04
800	PC lipids^d^	0.39	21	1.33	79	0.47	0.65	0.04
1000	PC lipids^d^	0.39	18	1.84	82	0.58	0.81	0.03
400	PC lipids^d^	0.05	33	1.46	67	0.72	0.96	0.08
600	PC lipids^d^	0.05	17	1.56	83	0.75	0.88	0.04
800	PC lipids^d^	0.05	33	1.35	67	0.73	0.93	0.06
1000	PC lipids^d^	0.05	26	1.51	74	0.60	0.82	0.07
400	Thy lipids^e^	0.39	6	2.34	94	0.53	0.65	0.06
600	Thy lipids^e^	0.39	6	1.70	94	0.51	0.58	0.02
800	Thy lipids^e^	0.39	14	1.25	86	0.47	0.56	0.11
1000	Thy lipids^e^	0.39	8	1.78	92	0.51	0.61	0.03

Fitted lifetime components (*τ*) are expressed in terms of their percentage amplitudes (A).

〈*τ*〉 is the amplitude-weight average lifetime, standard deviation shown, 8 measurements per sample (except for ^c^, *n* = 3, where signal/noise was very high).

Fitted z-parameter was always approx. 0.1–0.2% of (A1 + A2), is not discussed further. $\mathrm{Fluor}\left(t\right)=A1.e^{-t/\tau 1}+A2.e^{-t/\tau 2}+z$

a Denotes concentration of LHCII solution incubated with mica surface for 20 min followed by washing surface with copious buffer to remove unbound protein, except for (b).

b Control sample, lifetimes measured from 10 nM LHCII in high-detergent solution using a cuvette-based spectrophotometer, data from Fig. 1E (all other samples measured using FLIM).

c Control sample, lifetimes measured from 1000 nM LHCII in low-detergent solution *above* mica (~5 μm).

d Incubated with DOPC lipid vesicles (600 μM) for 20 min, following LHCII adsorption to mica.

e Incubated with thylakoid lipid vesicles (600 μM) for 20 min, following LHCII adsorption to mica.

f Excitation fluence was calculated from the measured average power, known repetition rate and pulse FWHM and estimated losses (for full details, see Supplementary Fig. S1).

### 3.4 Lipids cause a rearrangement of LHCII and a less-quenched system

**Fig. 4:**
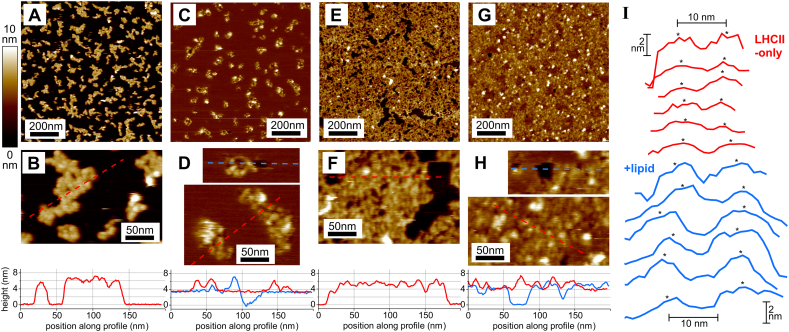
LHCII domains on mica before and after addition of lipids. (A) AFM topograph of aggregated LHCII domains (deposited at 600 nM for 20 min, washed). (C) AFM topograph of a parallel sample to (A), but after incubation with DOPC lipid vesicles (600 μM for 20 min) and gentle washing. (E) AFM topograph of aggregated LHCII domains (deposited at 1000 nM for 20 min, washed with salts). (G) AFM topograph of a parallel sample to (E), but after incubation with thylakoid lipid vesicles (600 μM for 20 min) and gentle washing. (B), (D), (F), (H) Higher magnification regions of (A), (C), (E), and (G), respectively, with height profiles (*below*). (I) Series of height profiles across representative LHCII protrusions. *Red:* across LHCII in aggregates from (A); or *blue:* across LHCII in DOPC SLBs from (C). (For interpretation of the references to color in this figure legend, the reader is referred to the web version of this article.)

We investigated the way in which LHCII aggregates are affected by lipids, as these molecules form the natural biological environment for membrane proteins. Furthermore, supported lipid bilayers (SLBs) on glass or mica are an established system for the dynamic study of simplified model membranes [49]. The common phospholipid dioleoyl phosphocholine (DOPC) (net-neutral, zwitterionic) was initially used in the current study because it forms high-quality bilayers with laterally-mobile lipids on hydrophilic substrates [50], and the physical chemistry of this process is well characterized [51]. Parallel samples of LHCII deposited onto mica at a moderate concentration (600 nM) were studied, either with or without incubation with lipid vesicles (samples were washed with fresh buffer prior to imaging, in either case). LHCII-only samples showed 2-D aggregate structures comparable to previous experiments (Fig. 4A–B), whereas samples incubated with lipid vesicles showed major differences likely interpreted as lipid bilayer formation around LHCII (Fig. 4C–D). Firstly, the majority of protrusions were only 1–2 nm above the immediately obvious surface, which correlates to extrinsic regions of LHCII protruding above the lipid bilayer. The lipid bilayer appears highly contiguous with very occasional holes found every few um^2^ (as is commonly reported for SLBs formed using this vesicle rupture technique). The height of the bilayer relative to the mica support was measured as ~4 nm at these defects (Fig. 4D, *blue height profile*), as expected for DOPC [50], therefore LHCII occupied a total height of 5–6 nm, in agreement with our AFM measurements without lipids. This confirmed that LHCII was successfully incorporated into the lipid bilayer.

Secondly, at a qualitative level there appeared to be more space intervening between neighbouring LHCII and some LHCII particles were clearly separated by over 20 nm. AFM analysis of these sample including lipids was more challenging with some poorly defined particles which may relate to a mobile population of LHCII (AFM scanning will produce “streaky” protrusions for moving particles). The trend for greater separation between LHCII in lipid-containing samples was apparent from height profiles drawn across representative pairs of LHCII (Fig. 4I). To substantiate this we performed a manual analysis of estimated centre-to-centre distance measured between nearest neighbour LHCII particles (see Table 2 and Supplementary Fig. S6 for further details). This confirmed a significantly greater average separation between LHCII due to the presence of lipids (13.5 ± 2.1 versus 10.5 ± 2.2 nm). Whilst there is a degree of uncertainty in the absolute values due to any errors in AFM scanning, the relatively broad S.D.s likely reflect genuine heterogeneity of LHCII separation. Thirdly, the fraction of the substrate surface area occupied by LHCII was significantly lower in the sample containing lipids (14% vs 28%, see Table 2 and Supplementary Fig. S7). This is likely to be due to lipid vesicles acting as a surfactant and removing some LHCII during the process of lipids diffusing around the membrane protein and causing rearrangements. Washing the sample with detergent-free buffer at the end of the lipid vesicle incubation period before imaging may halt the process of rearrangements by removing the source of excess lipids. This third effect may limit the accuracy of defining the protein-to-lipid ratio and reproducibility between samples.

**Table 2:** Analysis of AFM measurements comparing the effect of lipid membranes on LHCII organisation on mica.

Sample [LHCII] (nM)^a^	Lipids/DDM?	Surface coverage with LHCII ± S.D. (% total substrate area)^c^	Nearest neighbour LHCII distance ± S.D. (nm)^d^
600	None	27.9 ± 1.6	10.5 ± 2.2
600	Lipids^b^	14.2 ± 1.4	13.5 ± 2.1

a Denotes concentration of LHCII solution incubated with mica surface for 20 min followed by washing surface with copious buffer to remove unbound protein.

b Incubated with DOPC lipid vesicles for 20 min, following LHCII adsorption to mica.

c Mean of at least 4 AFM images taken of different regions of the substrate, standard deviation (S.D.) shown.

d Mean of well-resolved LHCII-LHCII pairs, S.D. shown (*n* = 49, 51, respectively).

A further series of experiments was performed using vesicles composed of natural thylakoid lipids monogalactosyl diacylglycerol (MGDG), digalactosyl diacylglycerol (DGDG), sulfoquinovosyl diacylglycerol (SQDG), phosphatidylglycerol (PG). Thylakoid lipids are less tractable as they do not form normal supported lipid bilayers on planar substrates due to the high content of the non-bilayer (high curvature) lipid MGDG [52]. Several preliminary experiments were performed to optimize the lipid composition and buffer conditions for the thylakoid liposomes in order to achieve a high-quality, contiguous supported lipid bilayer comparable to those of 100% DOPC. After initial tests with either the native mixture of thylakoid lipids or 100% DGDG were unsuccessful, 25% DOPC was incorporated into the thylakoid lipid mixture to promote formation of planar bilayers [44] which are amenable to surface-based work. This gave a final adapted mixture comprised 35% MGDG, 20% DGDG, 12% SQDG, 8% Soy PG, 25% DOPC (w/w%). These lipids appeared to be well mixed on a surface (and are thus available to interact with LHCII) as suggested by the observation that these membranes have excellent lateral lipid mobility and homogenous appearance both at the microscale (by fluorescence microscopy, see Supplementary Fig. S8) and nanoscale (by AFM, see Fig. 4G–H), with no evidence of lipid phase segregation. The buffer used in the lipid incubation stage was adapted to contain 5 mM CaCl_2_ to support the association of charged lipids with the substrate (see Supplementary information). We note that divalent cations could affect LHCII aggregation, but we do not observe any evidence of adverse effects.

Using the thylakoid-optimized protocol, we performed parallel experiments on LHCII aggregates, before and after thylakoid lipid vesicle addition. AFM data showed a higher initial surface coverage of aggregated LHCII (Fig. 4E–F) but after thylakoid liposome addition we observed the same trend as described for DOPC lipids: reduced surface coverage of LHCII (Fig. 4G–H) and a significant spacing out of LHCII protrusions. We did not attempt a quantitative analysis of AFM data from the thylakoid lipid membranes as the resolution did not appear as high as for DOPC, probably due to the more challenging nature of the sample. We conclude that the effect of thylakoid lipids is comparable to that observed for DOPC, showing no significant difference due to lipid type.

Fluorescence microscopy of lipid-containing LHCII on mica samples at a range of LHCII surface densities revealed significant differences to those without lipids, firstly, a significant increase in the intensity of fluorescence, approximately 5–10 fold higher with lipids versus without lipids. We did not attempt to quantify the absolute fluorescence counts because slight differences in the thickness of mica substrates may attenuate the fluorescence intensity to different degrees; instead we focussed on fluorescence lifetime data analysis which is not biased by signal intensity. A second qualitative difference was the observation of small numbers of brighter fluorescent spots which appeared to be mobile (Fig. 3A, *LHCII* (*DOPC*)). These are likely to be lipid vesicles associated (possibly tethered) to the membrane surface, as commonly observed in studies on lipid-only bilayers [50]. As the lipids used are not fluorescent, these surface-associated vesicles must contain LHCII lifted from the surface. We wished to correlate fluorescence data with the AFM mapping, which may not resolve these mobile structures; therefore, only stable regions of homogenous fluorescence intensity were analysed.

Emission spectra of these LHCII on mica samples in the presence of lipids showed the expected LHCII peak shape and maximum at ~681 nm, very similar to the spectra recorded for aggregates, with only minor peak broadening (Fig. 3B). Thus, the vast majority of protein is intact and in a native-like lipid environment. Picosecond fluorescence lifetime measurements on these samples revealed that lipid addition had indeed reduced the level of quenching by LHCII, manifested here as a slower decay compared to LHCII aggregates (see Fig. 3C, *arrow 2*), which is intermediate compared to LHCII solubilized in DDM. The average lifetime for lipid-LHCII samples was significantly higher at 〈*τ*〉 = 0.65–0.96 ns (DOPC) and 〈*τ*〉 = 0.56–0.65 ns (thylakoid lipids) compared to LHCII aggregates at 〈*τ*〉 = 0.20–0.29 ns (see Table 1), in agreement with the increased fluorescence intensity noted above. LHCII-lipid samples analysed with moderate fluence (0.39 mJ/cm^2^) compared to low fluence (0.05 mJ/cm^2^) had slightly reduced 〈*τ*〉 suggesting a subtle effect of potential singlet-singlet annihilation effects (0.65–0.86 ns compared to 0.82–0.96 ns). Annihilation effects due to high excitation power were further considered in the Supplementary information (see Fig. S1).

There did not appear to be a trend between different LHCII concentrations for lipid-containing samples (see Fig. 3D) and any differences are expected to reflect inconsistencies between lipid vesicle surfactant effects and washing processes which may be challenging to standardize (i.e. controlling the fluid dynamics caused by pipetting). Irrespective of sample to sample differences and (with or without) potential annihilation effects causing variation in absolute values, several repeated sample sets confirmed that the lifetimes observed after lipid addition were consistently significantly higher than before, see Fig. 3D. For additional fluorescence decay curves and emission spectra confirming these trends see Supplementary Figs. S4 and S5. Considering the separate lifetime components (Table 1): whilst LHCII aggregates were dominated almost entirely by the fast component *τ*2 (0.25 ns) with A2 > 95%, LHCII incorporated within lipid membranes had a lesser amplitude contribution A2 = 80–95% of fast component *τ*2 (0.4–0.6 ns) and A1 = 5–20% contribution of the slower component *τ*1 (1.3–2.0 ns) which suggests two sub-populations of LHCII in different quenched states, similar to the two states found for LHCII in DDM with roughly 50% contribution each from the fast and slow components (at *τ*2–1.2 ns and *τ*1 > 3 ns).

## 4 Discussion

### 4.1 Observation of quenched LHCII aggregates vs less-quenched LHCII in membranes

**Fig. 5:**
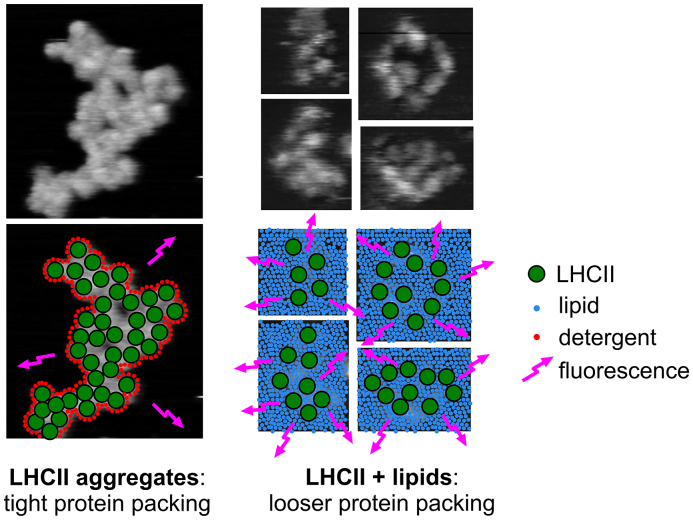
Schematic of possible arrangements of LHCII on mica. Cartoon representation showing how LHCII in 2-D aggregates on a surface (with a possible detergent perimeter) has low fluorescence (quenched state) because of self-associations due to tight packing, in comparison to the greater LHCII separation we observe when in a lipid bilayer and greater fluorescence (less quenched). These interactions are likely to be driven by thermodynamics and the hydrophobic effect, shielding apolar regions of the protein from water. These AFM images (adapted from Fig. 4, all to scale) were acquired on the same day with the same AFM probe and are therefore highly comparable and “more space” around LHCII is apparent with lipids. Positions of LHCII were estimated for this cartoon based on space-filling requirements where AFM resolution is imperfect.

Aggregates of LHCII, the commonly studied in vitro model for NPQ, were directly visualized as tightly-packed 2-D assemblies on a mica substrate by AFM with single-protein resolution and optically characterized by FLIM. We show that within this highly tractable system LHCII quenching may be manipulated through the simple addition of lipids, with reduced LHCII-LHCII interactions promoting a more fluorescent state of the complex (see schematic representation, Fig. 5). AFM showed that the mica surface area % coverage with LHCII could be controlled by varying concentration of LHCII in the incubation solution, but that LHCII always associated into domains (Fig. 2). After subsequent incubation with lipid vesicles, AFM revealed that LHCII incorporated into lipid membranes and rearranged into a more widely-spaced configuration (Fig. 4, Table 2). FLIM on parallel samples (Fig. 3, Table 1) showed that LHCII fluorescence lifetime decreased as follows: LHCII (in detergent solution) > LHCII (in lipid membranes on mica) > LHCII (aggregates on mica). The effects were very similar whether considering a model bilayer of phospholipids or the natural thylakoid lipids. Singlet-singlet annihilation effects did not affect this trend but may decrease lifetimes by 10–30% (shown by variation of excitation fluence Figs. 3D, S1). To our knowledge, this is the first in vitro correlation of high resolution protein arrangement (via AFM) to the quenched state (via FLIM) of just a few LHCII assemblies. We conclude that lipids cause important changes in vitro that may be important within the natural thylakoid membrane, namely that lipids modulate the extent of LHCII-LHCII interactions and therefore the propensity for the complex to activate NPQ.

### 4.2 Advantages of the mica substrate and the biological importance of the lipid environment for FLIM/AFM

Muscovite mica is a sheet silicate that can be cleaved to reveal a near-perfectly flat hydrophilic surface and has long been used as an ideal support for samples in AFM studies, including the majority of high-resolution studies on LH membranes [[53], [54], [55], [56], [57], [58]] and on phase-segregating SLBs [59]. However, mica is not ideal for optical microscopy as it not entirely transparent, nor rigid, thus glass is a more common substrate for optical microscopy for ease-of-use considerations. Furthermore, the surface chemistry of cleaved mica is not straightforward [60], whereas silane chemistry for functionalization of borosilicate glass is well-established. Vasilev et al. [28] demonstrated a novel protocol for nanoscale array patterning of LHCII, with covalent attachment of the protein to glass. The authors showed that detergent induced reversible switching of surface-bound LHCII between quenched and nonquenched states. In the current study we take an alternative approach by using thinly-cleaved mica as a flat substrate which provides a non-specific, non-covalent ionic association to the protein/lipid materials and is amenable to high resolution AFM. This substrate promoted the controlled self-assembly of LHCII and apparent confinement in two dimensions producing single-layer protein domains (Fig. 2), as compared to the large 3-D aggregates assumed to occur when detergent is removed from LHCII in aqueous solution [25, 26]. Note that if we instead used a hydrophilic glass coverslip as a substrate (without any silanization) very little LHCII was found on the surface by AFM or fluorescence microscopy (data not shown). We could control the fraction of the mica surface covered with LHCII into either isolated relatively-small 25–100 nm width patches or a densely packed network (Fig. 2). The fact that across all measured LHCII surface coverages we found that the degree of quenching is similar (Table 1) confirms that long-range energy transfer between many LHCII is not critical for quenching in aggregates. Thus, controllable 2-D cluster formation of LHCII on mica allowed correlation of spatial information of AFM that is crucial to confirm the protein arrangement for clear interpretation of functional information gained by optical imaging.

The fact that protein (and lipid) association with the surface is non-covalent allows rearrangement or removal of components which may be desirable or undesirable. It is well-known that lipid bilayers supported on glass or mica (SLBs) are fluid [49], i.e. lateral diffusion of lipid molecules occurs, due to a 0.5 nm thick water layer spontaneously formed between the substrate and during lipid bilayer self-assembly [61]. We note that SLBs of both DOPC and the thylakoid lipid mixture showed high fluidity on mica (Fig. S8) expected for high quality contiguous membranes. We should also consider the lateral diffusion (or not) of LHCII. On mica LHCII in 2-D aggregates appears to be immobile due to its interaction with the substrate (Fig. 2). This could be modulated in future studies by use of a polymeric cushion layer to allow membrane protein diffusion [62]. The technical difficulty that we found when imaging LHCII samples after lipid membrane formation by AFM (Fig. 4, streaky/blurred regions of images) could hint that some of the LHCII that remains within the lipid bilayer is now laterally-mobile, but this is yet to be confirmed. Another consideration is the orientation of LHCII, which may be 50/50% “up”/“down”, compared to the specific luminal/stromal orientation of LHCII in natural thylakoids. Alternatively, LHCII orientation can be controlled by crosslinking of specific residues to the substrate [28], although this would disallow protein diffusion. A challenge of the transient nature of the LHCII-mica interaction is that we found a portion of LHCII was removed from the surface by lipid vesicles (manifested as a reduced surface area coverage, Table 2), which may limit the consistency between samples. If the laterally-mobile LHCII could be controlled this would be relevant for the biological situation, as LHCII rearrangements within natural thylakoid membrane are involved in NPQ [22].

Detergents are often suggested to mimic the native environment of transmembrane proteins, but single-chain surfactants are clearly very different to lipids and may stabilize various non-native protein conformations. We have demonstrated an intermediate level of complexity where a native-like membrane environment can be generated by addition of lipid vesicles to pre-formed protein clusters and protein-protein/lipid communication is allowed, the same logic as applied in proteoliposome studies, but with the advantage of depositing the samples on a planar support allowing high-resolution microscopy of AFM and FLIM. By extension, the lipid membrane environment also allows the option of inclusion of membrane-soluble cofactors of relevance to photosynthesis, for example, a recent study constructed in vitro multi-layer membrane with quinones diffusing between enzymes [63]. Further, there is the potential to extract and introduce new components into any fluid SLB to modulate the membrane composition [64] or force the direct membrane insertion of small membrane proteins from detergent solution [65]. Developing a surface-based controllable membrane could provide the flexibility to directly visualize the effects of LHC and photosystem protein diffusion and rearrangement [22, 40] or the effect of PsbS diffusing through the membrane to LHCII [66].

### 4.3 Discussion of the exciton dynamics within LHCII

The protein scaffold of LHCII configures its bound pigments precisely with chlorophyll-chlorophyll *a*nd chlorophyll-carotenoid separations of 10–15 Å [4], leading to rapid energy transfer and within one monomer (intra-monomer) and spatial equilibration across the trimeric LHCII complex (including somewhat slower inter-monomer transfers) likely to occur in tens of ps [45, 47, 67]. Thus, considering this relatively fast equilibration the overall lifetime represents the fastest dissipative pathway. In the emissive state of LHCII this is reflected as fluorescent decay rate similar to a typical single isolated Chl (lifetime ~4 ns) as energy is transferred to the terminal lower-energy Chl *a* pigments and emitted at approximately 681 nm, a broad peak due to subtle differences in the local environment around pigments. Alternatively, in the quenched state of LHCII a faster non-radiative dissipative pathway occurs and there is less fluorescence (with lifetime <1 ns). Our sequential AFM/FLIM measurements provide protein mapping with 1–2 nm lateral resolution and optical information of a sub-micron region of interest (ensemble of ~100–500 LHCII within the focussed laser spot) with temporal resolution for fluorescence lifetimes of ~200 ps. As expected, we observe these signatures characteristic of highly-emissive LHCII emission with 〈*τ*〉 = 2–4 ns in detergent solution, whereas, when 2-D aggregates of LHCII assemble the lifetime is greatly decreased to 〈*τ*〉 = 0.2–0.29 ns, approximately at our temporal resolution limit (Fig. 3, Table 1). Multi-exponential decay fits suggest that each exponential represents a different decay pathway. The two lifetime components we observed are expected to represent two separate states of LHCII each with a single dissipative route rather than two dissipative pathways per LHCII, as proposed by others [27], due to above-mentioned very rapid intra-complex energy equilibration. In agreement with these studies, we observe by FLIM that both the long and short lifetime component of LHCII are concomitantly reduced from detergent to lipids to aggregates, as the average distribution of each state we observe gets progressively quenched. Cuvette-based spectrometers make an ensemble measurement of billions of complexes therefore may miss rare events, but can achieve higher temporal resolution of <25 ps due to higher signal-to-noise via longer collection times and narrower IRF. A previous report of aggregated LHCII trimers in solution concluded an average of one LHCII in a highly-quenched state per 4 LHCII trimers (〈*τ*〉 of 190 ps including an extremely short at 25–40 ps decay component and several longer components) [68]. Our observations where just a few LHCII are averaged reveal 2-D clusters ranging from 25 to 150 nm width and containing from 5 to 50 LHCII trimers and average lifetime of ~250 ps, supporting this conclusion.

Note that whilst we observed dramatic fluorescence intensity and lifetime changes, the shape of the LHCII emission peak was largely unchanged and positioned at 681–682 nm in our FLIM of LHCII whether in solution, in aggregates or in lipid membranes (Fig. 3). Recent single molecule spectroscopy of isolated complexes has shown that LHCII spontaneously switches between the nonquenched and multiple quenched states due to the inherent protein conformational disorder of LHCII [30], the latter having characteristic lower energy (red) states of LHCII previously only detectable under cryogenic temperatures as fluorescence peaking at 700 nm [36]. Importantly, the known NPQ-inducing conditions of pH and Zea simply shifted the population to access the quenched state for a greater time [31, 32]. These single-molecule studies proposed a three state model between a nonquenched state (high 681-nm emission and long lifetime) and two quenched states (low emission at 700 or >760 nm, short lifetime). The molecular mechanism for energy dissipation in the NPQ state is that additional dissipative pathways become accessible due to subtle conformational shifts of the protein changing the pigment configuration, bringing terminal Chls critically closer to low-energy carotenoids, potentially involving charge-transfer (CT) states or hybrid mixed CT-exciton states, leading finally to non-radiative energy dissipation from the S1 state of carotenoids as heat [16, 17, 33, 34]. Kruger and van Grondelle recently reviewed differences between quenching due to high pigment concentration, aggregates and photoprotection in NPQ [69].

In the current study, we likely do not observe the red-shifted emission spectra of low-energy states because our FLIM system measures the ensemble of the >100 complexes within the focused laser spot and the likelihood that these NPQ-states have low fluorescence intensity and are only accessed for 1–2% of the time [31]. Thus, we simply state that our FLIM measurements are in full agreement with previous bulk spectroscopy measurements of NPQ, and, whilst our experiments do not currently observe them, current NPQ mechanistic proposals appear reasonable. We expect that protein-protein interactions which are strong within aggregates but are limited by the introduction of lipids (Fig. 5), are another effect that shifts the population towards accessing an NPQ-like conformation. In order to observe the low-signal low-occupancy red states of quenched complexes in future LHCII/lipid/mica FLIM experiments, one could study LHCII at a sufficiently low dilution within the lipid membranes that on average there is only 1 complex present within the laser spot (the strategy employed by single molecule studies), or alternatively, very high spatial optical resolution (<10 nm) would be required to resolve the densely-packed complexes. The former is challenging for our set-up to collect sufficient signal and the latter would be highly technically challenging requiring super-resolution detection of native chromophores (rather than idealized fluorophores designed for super-resolution applications). We have shown proof-of-concept for the utility of nanoscale topography and optical detection of low-number of complexes for observing NPQ switching, we suggest that extending this combinatorial approach to also incorporate metal nanoparticles for localized enhancement of excitation or emission [70] could allow the ultimate in vitro platform for mapping how NPQ changes and exciton dynamics change within native-like membrane environments with single-protein optical and topographic resolution.

### 4.4 Challenges and advantages of the thylakoid lipid system

The natural lipids of thylakoid membranes are unusual galactolipids, and these provide the most realistic mimic of the natural membrane environment, rather than common phospholipids. The most prevalent thylakoid lipid, monogalactosyldiacylglycerol (MGDG), has a high degree of negative curvature and is essential for correct thylakoid multi-layer stacking [71]. LHCII requires the bound lipids DGDG and PG to maintain its trimeric form [4, 72] and a membrane environment including MGDG and DGDG increases the stability and activity of LHCII and *PSI*I [73, 74]. The thylakoid membrane environment will also be important for any process which requires in-membrane protein-protein interactions e.g., PsbS [66]. However, the highly-curved lipid MGDG is challenging to work with as it forms inverse hexagonal (HII) phases [52]. Proteoliposomes containing LHCII and thylakoid lipids are stable due to the inherent curvature of a vesicle and are becoming the standard for in vitro studies of NPQ in solution [27, 40, 41, 43, 66, 75, 76], however, proteoliposomes are not conducive to high resolution imaging. Few studies have achieved the formation of planar bilayers of thylakoid lipids on a solid support as required for high resolution microscopy, due to the high curvature of MGDG [44]. We have demonstrated that it is possible to generate membranes including LHCII with thylakoid lipid mixtures on solid supports, and found similar protein rearrangements and similar intermediate levels of quenching as when using DOPC phospholipids (Fig. 3, Fig. 4; Table 1).

## 5 Conclusions

The experimental system we have demonstrated in which lipids and LHCII are assembled on a mica surface may be considered as a highly-simplified analogue to the natural thylakoid membrane system with the added advantage that planar domains of LHCII can be readily imaged with AFM, unlike highly curved liposomes, allowing nanoscale arrangement of complexes to be correlated with their functional properties. Indeed, the lifetime of the LHCII-lipid-mica system (〈*τ*〉 of 0.5–0.9 ns) parallels that found in liposomes and for native thylakoid membranes within chloroplasts: for example, Moya and co-workers found 〈*τ*〉 ~0.7 ns in proteoliposomes at high protein/lipid ratio [27] whereas Johnson and Ruban found 〈*τ*〉 from 0.6 to 1.6 ns, for chloroplasts undertaking NPQ in the presence of zeaxanthin [35]. This ‘poised’ state of LHCII found in lipid bilayers has been found to be more amenable to alterations in pH and xanthophyll content and so could provide a useful mimic of the in vivo system [66, 76]. The current study shows that the LHCII-lipid-mica system provides several advantages over common solution-based proteoliposome studies, but requires further refinement to mitigate the effect of lipids removing LHCII from the surface and to observe single-molecule mechanistic details. Future analysis of this system could include the addition of the PsbS protein, the xanthophylls zeaxanthin or violaxanthin and alteration of the pH in order to study their crucial NPQ-related effects in a controlled and traceable biologically-relevant membrane environment.

## Transparency document

Transparency document. Image 1

## Appendix A Supplementary data

Supplementary material - eight figures, one table, additional explanatory text. Image 2
