# Supplementary material for: Correlated fluorescence quenching and topographic mapping of Light-Harvesting Complex II within surface-assembled aggregates and lipid bilayers
Source: Biochim Biophys Acta. 2018 Oct;1859(10):1075–85. doi: 10.1016/j.bbabio.2018.06.011 (PMC6135645; doi:10.1016/j.bbabio.2018.06.011)
Supplement: Supplementary file 2 — Supplementary material - eight figures, one table, additional explanatory text. [file mmc2.docx]

**Supplementary Information for:**

**“Correlated fluorescence quenching and topographic mapping of Light-Harvesting Complex II within surface-assembled aggregates and lipid bilayers”**

Peter G. Adams ^a,b,*^, Cvetelin Vasilev ^c^, C. Neil Hunter ^c^, Matthew P. Johnson ^c^

^a^ School of Physics and Astronomy, University of Leeds, Leeds, LS2 9JT, UK.

^b^ Astbury Centre for Structural Molecular Biology, University of Leeds, Leeds, LS2 9JT, UK.

^c^ Dept. of Molecular Biology and Biotechnology, University of Sheffield, Sheffield, S10 2TN, UK.

**Fluorescence lifetime controls to test for singlet-singlet annihilation effects**

We varied the excitation fluence over a range expected to be above and below the level where singlet-singlet annihilation effects may occur. **Fig. S1** and **Table,** below, shows FLIM data acquired on a sample of LHCII aggregates on mica (*black squares*) or a sample of LHCII within DOPC lipid bilayers on mica (*red squares*). Multiple different regions from one sample were measured for a series of different excitation fluences. The fluence was modulated by a combination of different laser power settings and insertion of an ND filter (OD = 1) (as shown in the **Table**). A consistent repetition rate of 0.5 MHz and laser spot diameter of ~800 nm was used for all (pulse FWHM is estimated as 50 ps). The acquisition time was adapted to deliver approximately the same number of excitation photons during each measurement (as shown in the **Table**). A new IRF was measured at each new power setting.

The graph shows how the average lifetime <𝞽> of each sample varies with fluence (trend lines drawn to guide the eye). For LHCII aggregates (*black line*), there is no apparent difference in observed <𝞽> vs excitation fluence. For LHCII in lipid bilayers (*red line*), the observed <𝞽> decreases from ~0.85 ns at low excitation fluence to ~0.6 ns at low excitation fluence. The *dashed green* and *dashed blue* shows the “moderate fluence” and “low fluence” used to take measurements on series of LHCII concentrations in the main paper (Fig. 3). Moderate fluence is used to collect FLIM spectra at a reasonable speed and with high signal but is shown here to cause some effects due to singlet-singlet annihilation. Low fluence is used to avoid annihilation but collection time is longer and signal lower (we did not use lower fluence for the main paper because the signal-to-noise is low, reducing the reliability).


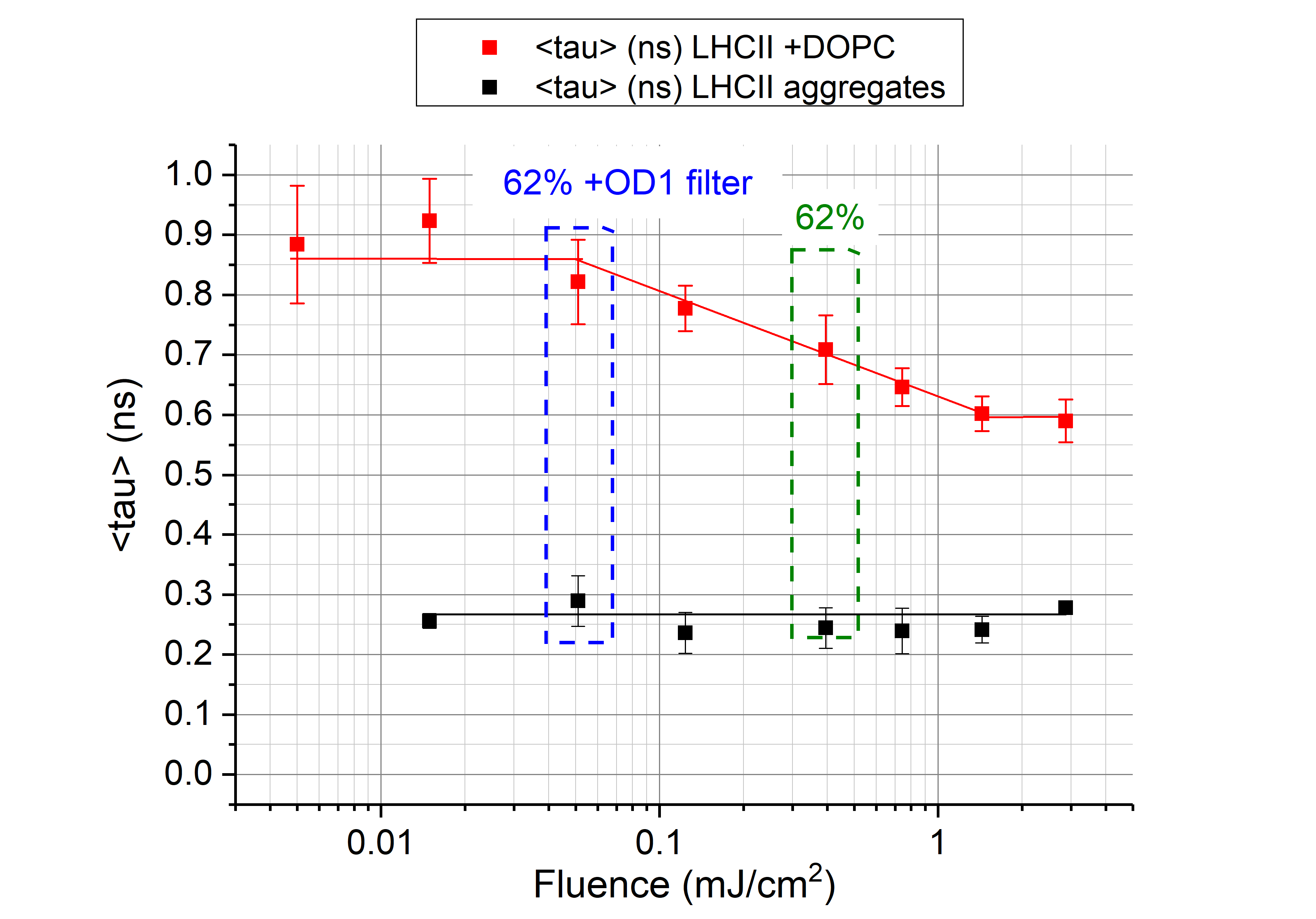


**Fig. S1 – average fluorescence lifetime versus excitation fluence**

| **LHCII conc (nM)** | **Lipids?** | **Laser  ± filter** | **Acq. time (s)** | **Measured av. power (W)** | **Excitation fluence (mJ/cm^2^)** | **A1 (%)** | **T1 (ns)** | **A2 (%)** | **T2 (ns)** | **<T> (ns)** | **<T> S.D. (ns)** |
| --- | --- | --- | --- | --- | --- | --- | --- | --- | --- | --- | --- |
| 1000 | none | 100.0% | 0.6 | 5.63E-05 | 2.867 | 32 | 0.30 | 68 | 0.27 | 0.28 | 0.01 |
| 1000 | none | 81.0% | 1.1 | 2.82E-05 | 1.436 | 15 | 0.38 | 85 | 0.23 | 0.24 | 0.02 |
| 1000 | none | 70.3% | 2.1 | 1.46E-05 | 0.744 | 9 | 0.44 | 91 | 0.22 | 0.24 | 0.04 |
| 1000 | none | 62.0% | 4.0 | 7.75E-06 | 0.395 | 20 | 0.35 | 80 | 0.22 | 0.24 | 0.03 |
| 1000 | none | 56.5% | 13.0 | 2.43E-06 | 0.124 | 25 | 0.51 | 75 | 0.19 | 0.24 | 0.03 |
| 1000 | none | 62% +ND | 31.0 | 1.00E-06 | 0.051 | 29 | 0.38 | 71 | 0.25 | 0.29 | 0.04 |
| 1000 | none | 56.5% +ND | 106.0 | 2.93E-07 | 0.015 | 47 | 0.33 | 53 | 0.19 | 0.26 | 0.01 |
| 1000 | none | 54.7% +ND | 316.0 | 2.93E-07 | 0.005 |  | N/A – signal too low for fit | | | |  |
| 1000 | PC lipids | 100.0% | 0.6 | 5.63E-05 | 2.867 | 8 | 1.65 | 92 | 0.50 | 0.59 | 0.04 |
| 1000 | PC lipids | 81.0% | 1.1 | 2.82E-05 | 1.436 | 10 | 1.57 | 90 | 0.50 | 0.60 | 0.03 |
| 1000 | PC lipids | 70.3% | 2.1 | 1.46E-05 | 0.744 | 12 | 1.55 | 88 | 0.53 | 0.65 | 0.03 |
| 1000 | PC lipids | 62.0% | 4.0 | 7.75E-06 | 0.395 | 15 | 1.56 | 85 | 0.56 | 0.71 | 0.06 |
| 1000 | PC lipids | 56.5% | 13.0 | 2.43E-06 | 0.124 | 20 | 1.50 | 80 | 0.60 | 0.78 | 0.04 |
| 1000 | PC lipids | 62% +ND | 31.0 | 1.00E-06 | 0.051 | 26 | 1.51 | 74 | 0.60 | 0.82 | 0.07 |
| 1000 | PC lipids | 56.5% +ND | 106.0 | 2.93E-07 | 0.015 | 34 | 1.47 | 66 | 0.66 | 0.92 | 0.07 |
| 1000 | PC lipids | 54.7% +ND | 316.0 | 9.80E-08 | 0.005 | 32 | 1.52 | 68 | 0.59 | 0.88 | 0.10 |

**Table – lifetime component analysis of the FLIM dataset with fluence varied.**

**Note on estimation of actual excitation fluence**

For full details, see spreadsheet in our raw data deposited online in the Research Data Leeds repository [1] under DOI: 10.5518/313. The average power was measured at the end of the multi-mode excitation fibre for each setting (after any ND filter). Thus, we calculated:

**excitation fluence [no losses]** = (peak power) x (pulse FWHM) / (laser spot area)

*where,* peak power = (measured average power) / (duty cycle)

*where,* duty cycle = (pulse FWHM) x (rep. rate)

Alternatively, an online calculator for peak power can be used, e.g. [www.calctool.org/CALC/phys/optics/pulsed_source](http://www.calctool.org/CALC/phys/optics/pulsed_source)

The excitation delivered to the sample is estimated to be ~12.8% of this due to losses in the system (multiplicative losses due to the fibre coupling, beam expander, beam combiner, dichroic mirror/ excitation filters, and objective of 50%, 20%, 50%, 20% and 20%, respectively).

*Thus,* **excitation fluence [after losses]** = (excitation fluence [no losses]) x 0.128

**Pulse energy** (in pJ) and **photons per pulse per unit area** is calculated simply from this, see “Excitation energy calculations” spreadsheet, here [1].

**Explanation of fluorescence emission spectroscopy**

**Fig. S2,** below shows cuvette-based measurements: an absorption spectrum (*blue*) compared to steady-state fluorescence emission spectra (*red*). The *vertical lines* (*blue*, *red*) relate the observed peaks to specific chlorophylls (chl). The *green vertical line* shows the laser excitation source. Intact LHCII trimers suspended at low concentration in 0.03% DDM (*solid curves*) vs denatured LHCII after treatment with 0.05% Triton X-100 (*solid curves*). After selective 485nm excitation of chl b we expect chl *b* -> chl *a* downhill energy transfer and a single peak of emission from chl *a* at 681 nm for intact LHCII trimers. Unfolding of the protein causes observation of significant emission of chl *b* at 653 nm (a double peak).


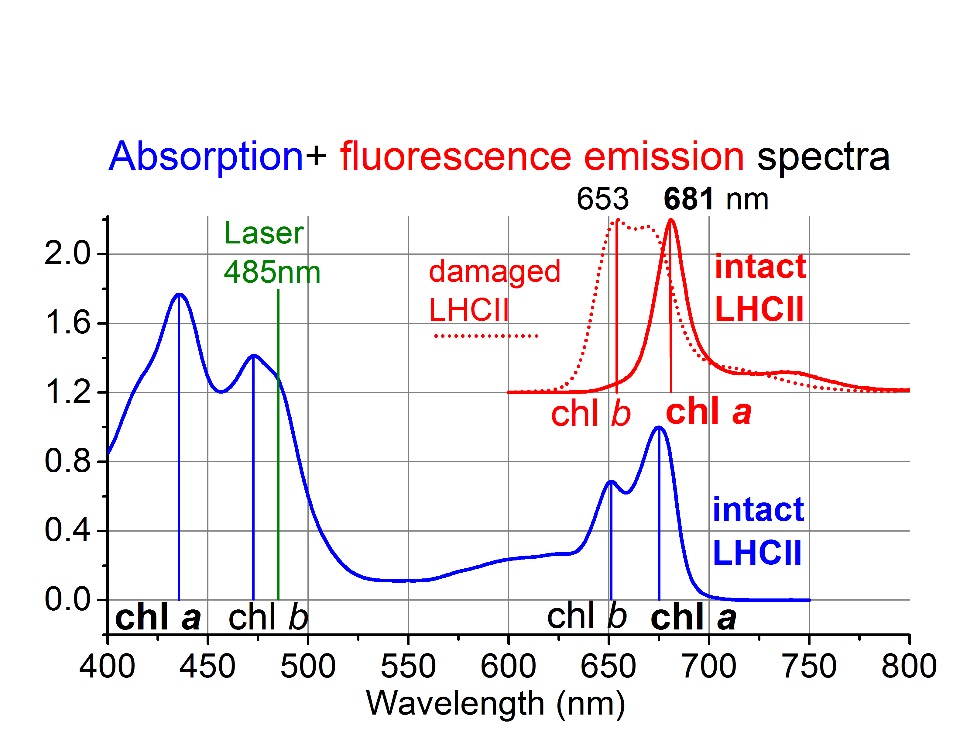


**Further AFM data on LHCII aggregates**

Further AFM images of LHCII aggregates on mica formed by incubation of the surface with the noted concentrations of LHCII in 20mM HEPES (pH 7.5), **Fig. S3,** below. These show a consistent organisation of LHCII complexes into domains and trend for increased surface with incubation concentration. Note, a control experiment of incubation of freshly-cleaved mica with 0.03% DDM in buffer leads to AFM observation of an apparently empty surface (data not shown), thus detergent alone does not form observable surface structures.


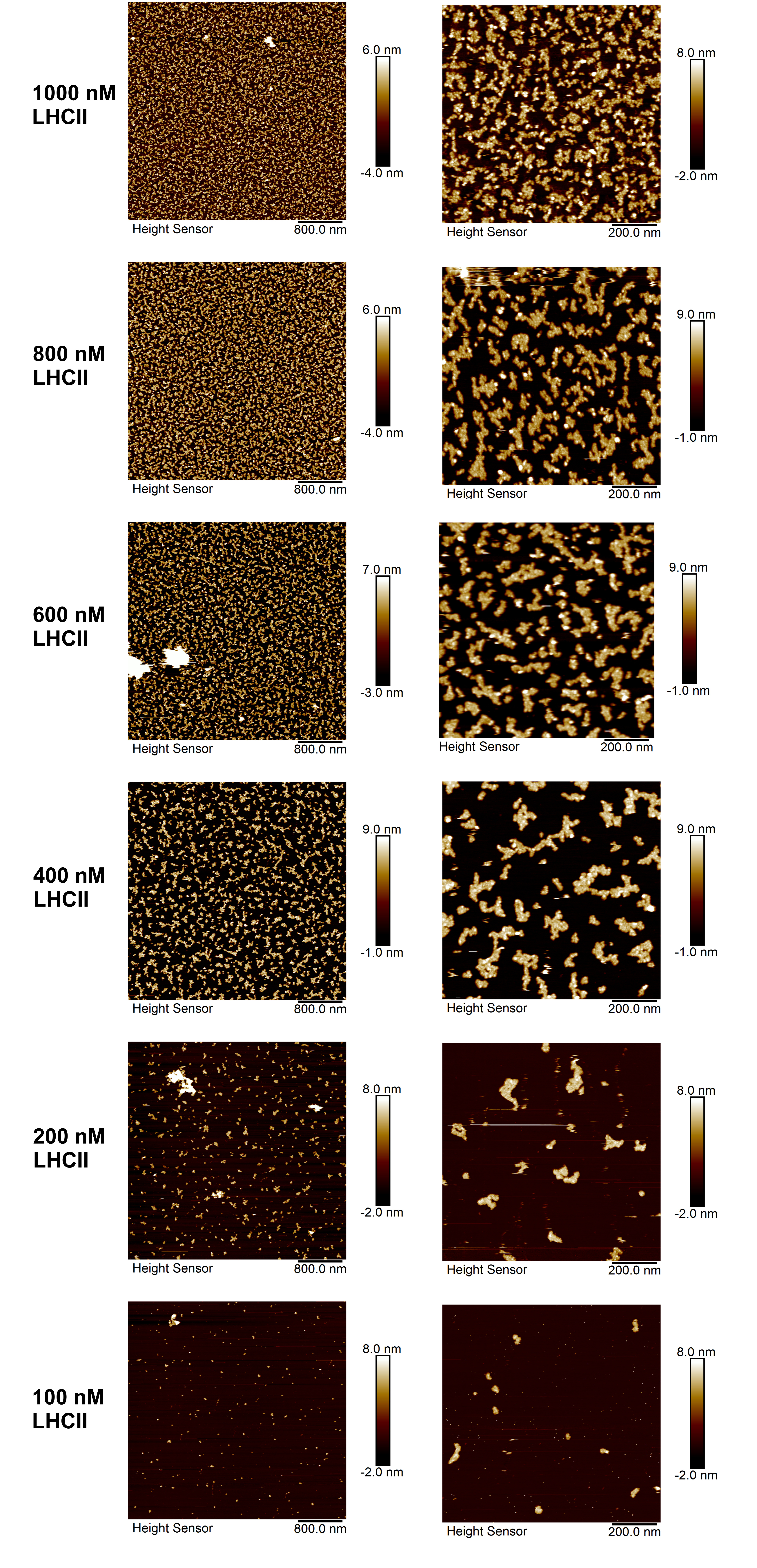


**Further fluorescence data**

All time-resolved and steady-state fluorescence raw data is freely available online in the Research Data Leeds repository [1] under DOI: 10.5518/313. Further fluorescence decay curves acquired from sample on mica using the FLIM system are shown in **Fig. S4**. This includes a representative decay curve from each LHCII concentration assessed (from main text Table 1 and Fig. 3) for (i) LHCII in detergent, (ii) LHCII aggregates (ii) LHCII in DOPC membranes and (iv) LHCII in thylakoid lipid membranes. These decay curves qualitatively show that LHCII aggregates have consistently very fast fluorescence decay (*green-brown curves*) and there is little obvious difference between concentrations 200-1000 nM compared to the slow decay of LHCII in DDM detergent (*red curve*). LHCII in DOPC membranes (*purple-pink* *curves*) have moderate-fast decay that are overlapping but as a population average are slightly faster than LHCII in DOPC membranes (*purple-pink* *curves*). Note, all curves have good signal-to-noise of these decay curves, only those highly quenched become noisy at ≤1% maximum counts (i.e. y≤ 0.01, here).


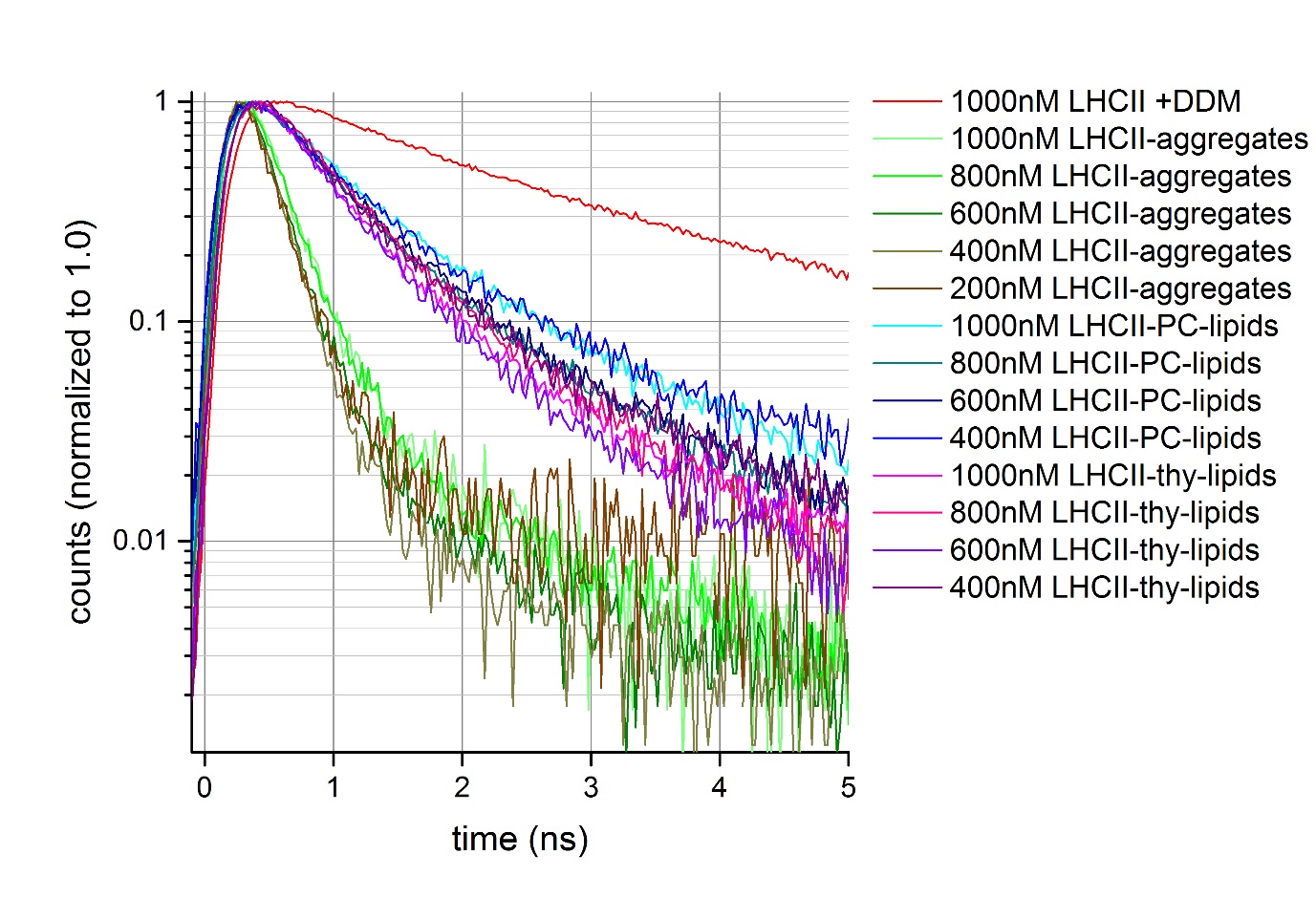


**Fig. S4 – representative time-resolved spectra before fitting, normalized to 1.0**

Further steady-state fluorescence emission curves acquired using the FLIM system are shown in **Fig. S5**, from samples on mica as in main text Table 1. A separate panel is shown for each sample type, for clarity to avoid too many overlapping curves. **Fig. S5A** shows LHCII aggregates have an emission peak with maxima at 681 nm, irrespective of LHCII concentration (400-1000 nM). These spectra are somewhat noisier than those from **Fig. S5B-C** because LHCII aggregates are so heavily quenched and thus there is less signal with similar acquisition parameters. **Fig. S5B** and **Fig. S5C** shows that LHCII after incorporation into membranes of 100% DOPC or the thylakoid lipid mixture, respectively, also have an emission peak with maxima at 681 nm. Some curves show no evidence of any change to the spectra, some samples have between 1-10% (DOPC) or 1-15% (thylakoids) contribution of a side peak at 665 nm. This does not appear to correlate with LHCII concentration nor with the lifetime fitted, thus we conclude that while there may be a few percent of the complexes with damage, this appears not to significantly affect the fluorescence lifetimes.


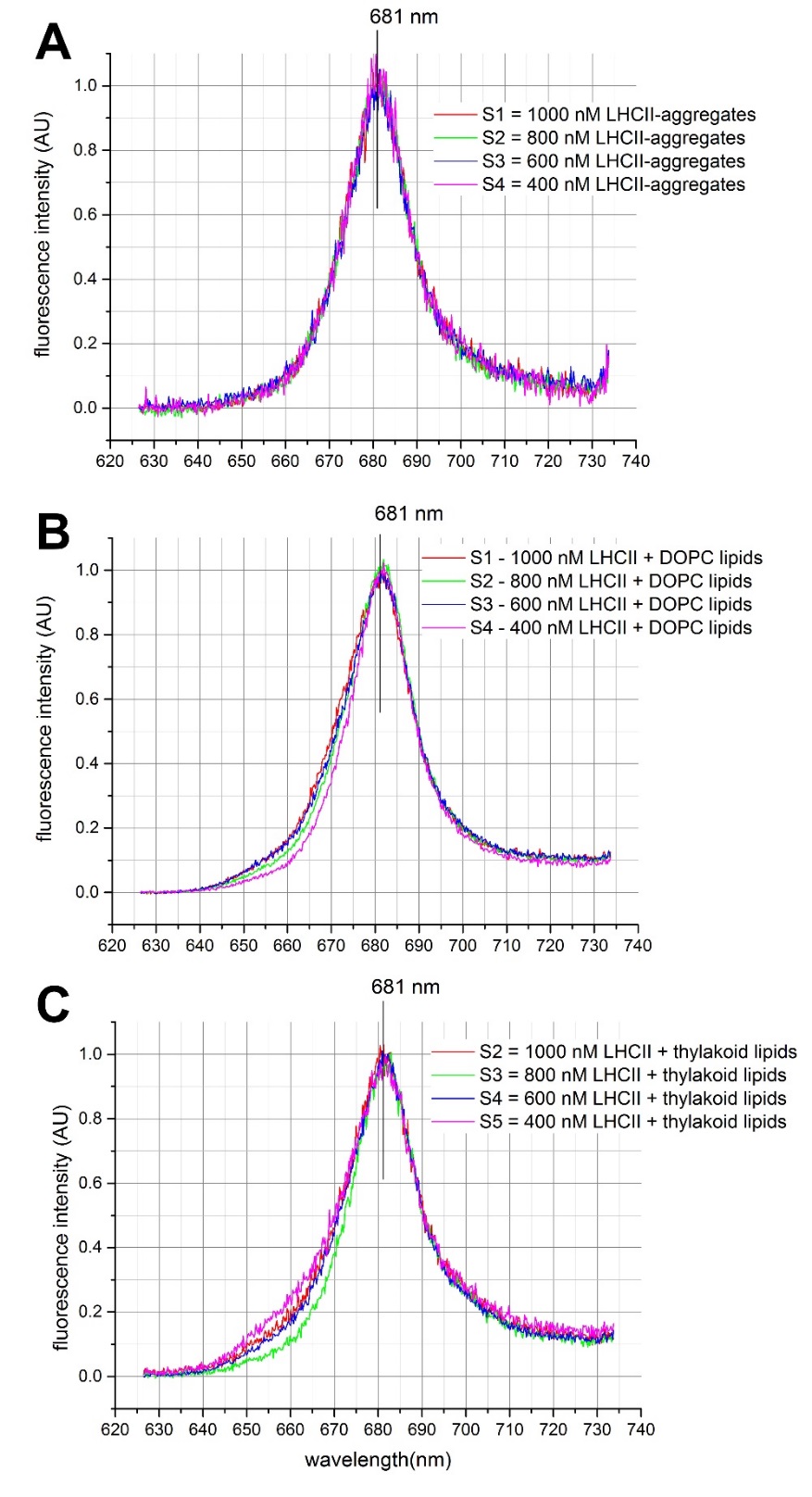


**Fig. S5 – representative fluorescence emission spectra, normalized to 1.0**

**Further AFM data showing LHCII-LHCII distances**

Images in **Fig. S6**, below, are AFM topographs showing the “LHCII-LHCII nearest neighbour” separation, i.e. measurement of protrusion centre-to-centre distance of LHCII particles, using Nanoscope Analysis software (provided by the instrument manufacturer, Bruker). Surface area analysis was performed using thresholding and area measurement operations in ImageJ software (Fiji package, open source), **Fig. S7**.


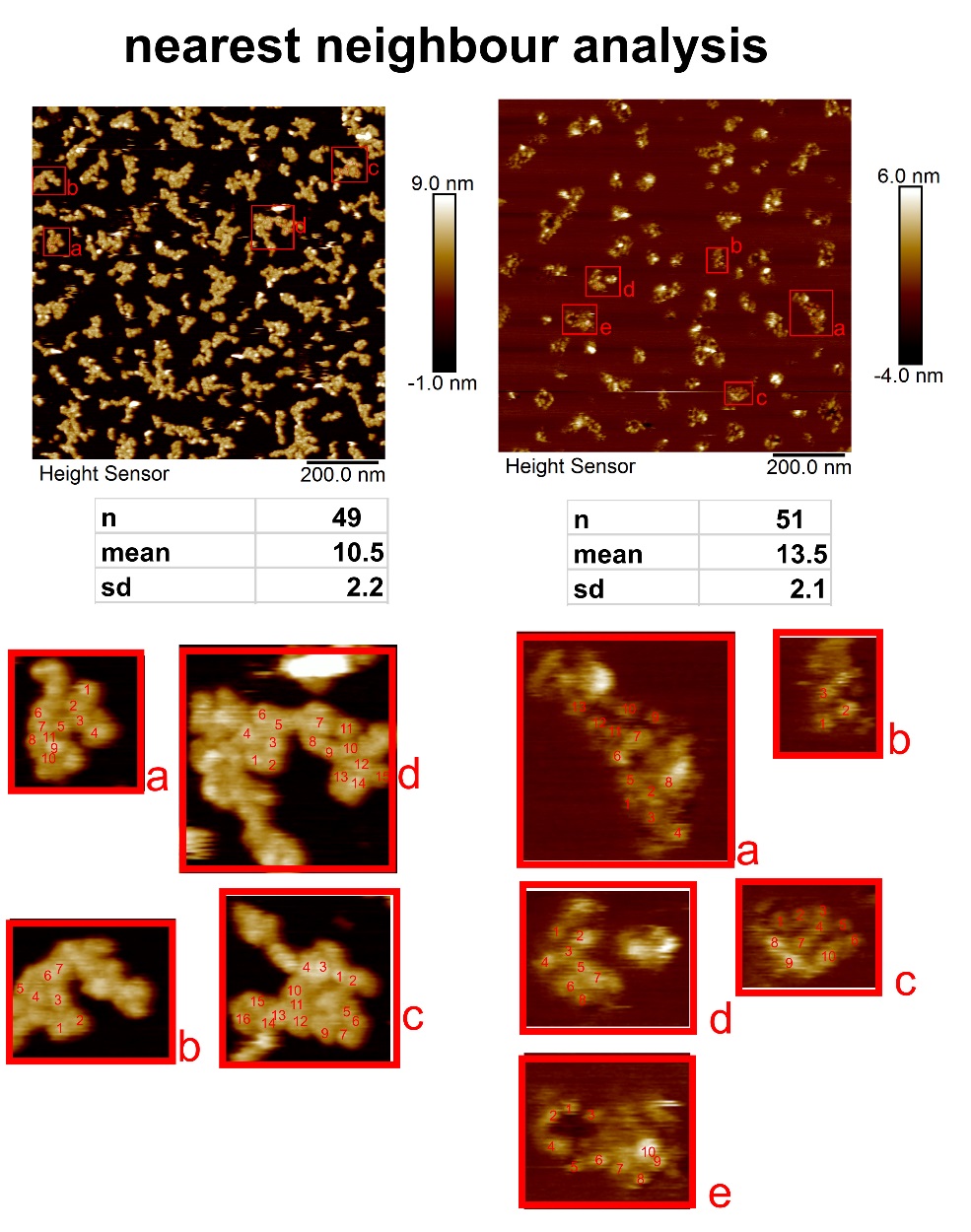


**Fig. S6 – Nearest neighbour analysis of LHCII on mica (left) and after DOPC vesicle incubation and washing (right).** Zoomed-in areas annotated, as noted on the image.


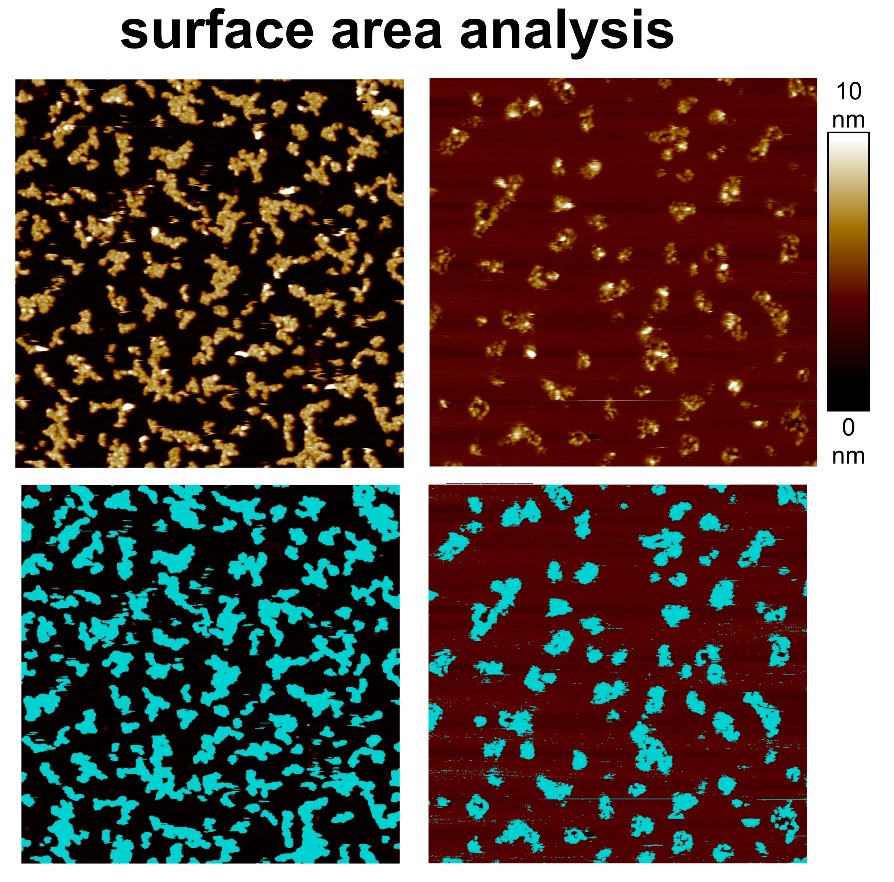


**Fig. S7 – Surface area analysis of LHCII on mica (*left*) and after DOPC vesicle incubation and washing (*right*)**

**Experiments with thylakoid lipids**

A series of experiments was performed to confirm the quality of the supported lipid bilayers (SLBs) formed from liposomes using either phospholipids (100% DOPC) or the thylakoid lipid mixture (comprising 35% MGDG, 20% DGDG, 12 % SQDG, 8% Soy PG, 25% DOPC w/v%). To allow membrane visualization by fluorescence microscopy (and in these experiments only), a fluorescently-tagged lipid Texas Red DHPE was additionally included at 0.5% (w/v). Fluorescence Recovery After Photobleaching (FRAP) measurements are a common way to measure the microscale lateral mobility of fluorescently-tagged lipids in the membrane [2]. Here, we calculate the diffusion coefficient (D) using the formula “D = 0.22.r^2^ / t_1/2_” (r = radius of bleached area; t_1/2_ = the recovery half-time measured from plots of corrected fluorescence intensity vs time). A high D is strong evidence for a high quality, contiguous SLB; low D suggests defects within the membrane (e.g. holes); zero recovery implies that the membrane is discontinuous (e.g. fragments of bilayers or intact vesicles).

Lipid vesicles were formed by probe sonication following rehydration of the desired composition of dry lipids into a particular buffer at a defined pH (20mM HEPES for pH 7.5; 20mM MES for pH 6.5 or 5.5) and supplemented with salt (150mM NaCl) or not. Vesicles were incubated with the substrate for 20-30 min and then washed with 10 changes of fresh buffer at the standard conditions 20mM HEPES, pH 7.5 (no additional salts) before imaging. A series of experiments were performed with samples of lipid vesicles which were prepared in different buffers, iteratively optimized in order to obtain bilayers with a high D. Conditions were first found for a piranha-cleaned (hydrophilic) glass coverslips as a substrate because working with glass is faster, and then this knowledge then applied to find optimal conditions for mica.

As can be observed in **Fig. S8** *rows 1-2*, DOPC SLBs formed on either glass or mica were highly fluid without requiring any additional supplements to the buffer, the lipid dye having a high D (0.6-0.9 µm^2^ s^-1^). As expected, the model lipid DOPC is highly amenable to various substrates and environments. The thylakoid lipids mixture used (“Thy-mix”) reflects a ratio similar to that in native spinach thylakoids [3], except containing 25% mol/mol of DOPC to improve its amenability to surface experiments, as suggested by Grab and co-workers [4]. Initial experiments showed that thylakoid lipids were challenging to work with (SLBs with low D), but we persevered to improve these because of their biological importance as the lipids present in the membranes in which LHCII natively resides. The thylakoid lipids formed very poor bilayers on glass at pH 7.5, see **Fig. S8** *row 3*, even with increased salt (to aid electrostatic screening of charges between negatively charged lipids SQDG and PG and the electronegative substrates). A lower pH and salts appeared to improve bilayer formation of the thylakoid lipids (see **Fig. S8** *row 4-5*) and at pH 6.5 and 5.5 the D was similar to DOPC, thus we proceeded with just pH 6.5. Mica as a substrate was more challenging for thylakoid lipids and poor results were observed for all pHs tested with monovalent salts (see **Fig. S8** *row 6*). Low concentrations of divalent cations have been previously found to improve vesicle fusion and spreading to, and we found that incubation for an additional 10 min with 5 mM CaCl_2_ allowed formation of the optimal bilayer for thylakoid lipids [5]. Thus, we used a buffer of 150 mM NaCl, 20mM MES, pH 6.5 followed by additional 5 mM CaCl_2_ incubation for all experiments with thylakoid lipids during the liposome incubation stage, exchanging back into our standard buffers at pH 7.5 for washing the sample prior to microscopy (including oxygen-scavenging enzyme and N_2_-sparging for all FLIM measurements).


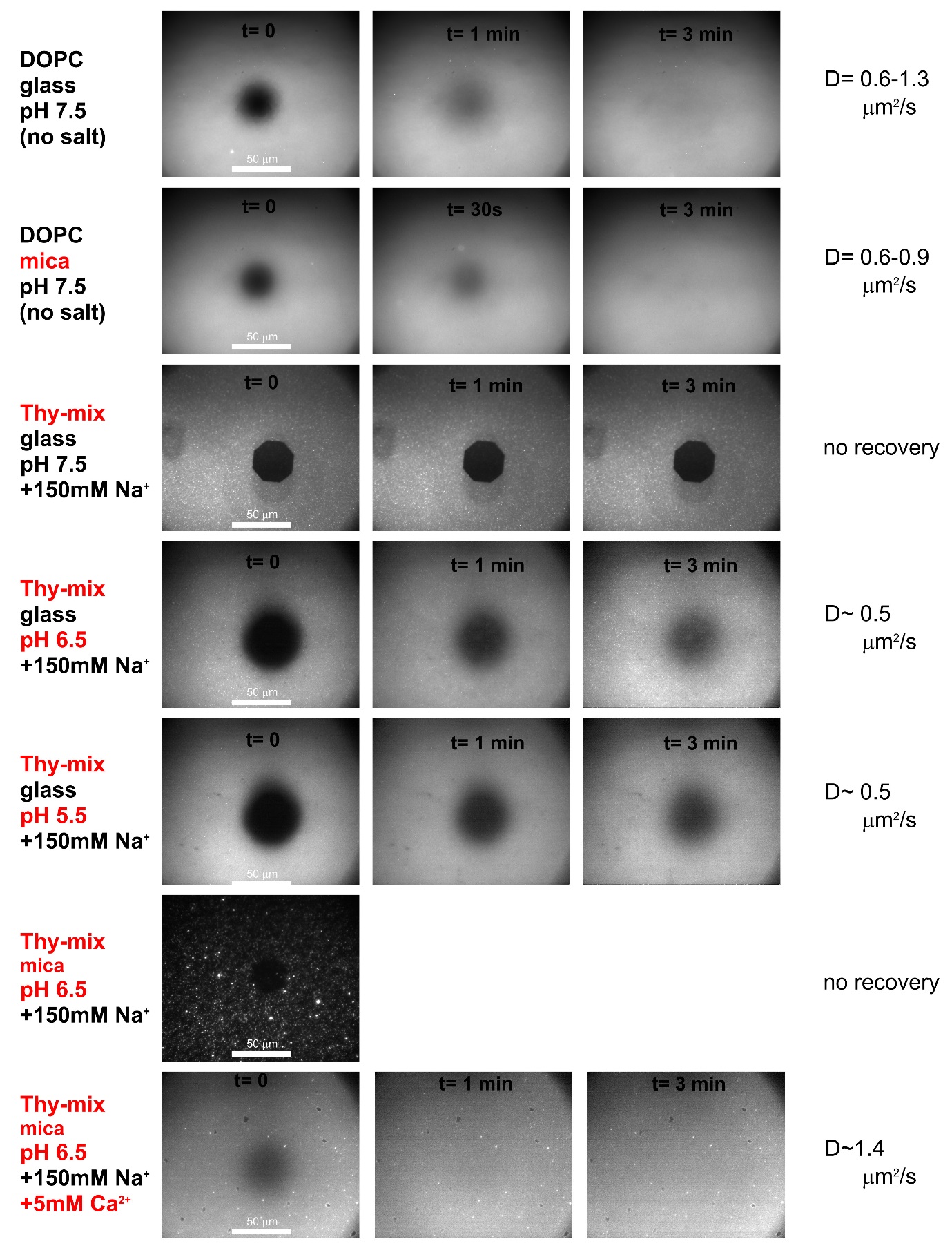


**Fig. S8 – Series of Fluorescence Recovery After Photobleaching (FRAP) measurements on different supported lipid bilayers (SLBs).** Fluorescence microscopy images of SLBs is shown immediately following photobleaching (t=0) and then at time points after this (t= 1 min, t= 3 min).

**References**

[1] P.G. Adams, C. Vasilev, C.N. Hunter, M.P. Johnson, Dataset for a study correlating fluorescence quenching and topographic mapping of LHCII aggregates and lipid bilayers, University of Leeds [Dataset] (2018) <https://doi.org/10.5518/313>.

[2] D. Axelrod, D.E. Koppel, J. Schlessinger, E. Elson, W.W. Webb, Mobility measurement by analysis of fluorescence photobleaching recovery kinetics, Biophys. J. 16 (1976) 1055-1069.

[3] C. Yang, S. Boggasch, W. Haase, H. Paulsen, Thermal stability of trimeric light-harvesting chlorophyll *a*/*b* complex (LHCIIb) in liposomes of thylakoid lipids, Biochim. Biophys. Acta, Bioenerg. 1757 (2006) 1642-1648.

[4] O. Gräb, M. Abacilar, F. Daus, A. Geyer, C. Steinem, 3D-membrane stacks on supported membranes composed of diatom lipids induced by long-chain polyamines, Langmuir 32 (2016) 10144-10152.

[5] D. Papahadjopoulos, S. Nir, N. Düzgünes, Molecular mechanisms of calcium-induced membrane fusion, J. Bioenerg. Biomembr. 22 (1990) 157-179.
